# *Valeriana jatamansi* Jones Inhibits Rotavirus-Induced Diarrhea via Phosphatidylinositol 3-Kinase/Protein Kinase B Signaling Pathway

**DOI:** 10.4014/jmb.2003.03006

**Published:** 2020-05-20

**Authors:** Bin Zhang, Yan Wang, Chunmao Jiang, Caihong Wu, Guangfu Guo, Xiaolan Chen, Shulei Qiu

**Affiliations:** 1 Jiangsu Agri-Animal Husbandry Vocational College, Taizhou, Jiangsu 225300, P.R. China; 2 Food, Animal and Plant Inspection and Quarantine Technical Center of Shanghai Customs, Shanghai 210000, P.R. China

**Keywords:** *Valeriana jatamansi* Jones, diarrhea, rotavirus, phosphatidylinositol 3-kinase/protein kinase B

## Abstract

Rotavirus (RV), as the main cause of diarrhea in children under 5 years, contributes to various childhood diseases. *Valeriana jatamansi* Jones is a traditional Chinese herb and possesses antiviral effects. In this study we investigated the potential mechanisms of *V. jatamansi* Jones in RV-induced diarrhea. MTT assay was performed to evaluate cell proliferation and the diarrhea mice model was constructed using SA11 infection. Mice were administered *V. jatamansi* Jones and ribavirin. Diarrhea score was used to evaluate the treatment effect. The enzyme-linked immunosorbent assay was performed to detect the level of cytokines. Western blot and quantitative reverse transcription-PCR were used to determine protein and mRNA levels, respectively. Hematoxylin-eosin staining was applied to detect the pathological change of the small intestine. TdT-mediated dUTP nick-end labeling was conducted to determine the apoptosis rate. The results showed *V. jatamansi* Jones promoted MA104 proliferation. *V. jatamansi* Jones downregulated phosphatidylinositol 3-kinase (PI3K) and protein kinase B (AKT) in protein level, which was consistent with the immunohistochemistry results. Moreover, *V. jatamansi* Jones combined with ribavirin regulated interleukin-1β (IL-1β), interferon γ, IL-6, tumor necrosis factor α, and IL-10, and suppressed secretory immunoglobulin A secretion to remove viruses and inhibit dehydration. *V. jatamansi* Jones + ribavirin facilitated the apoptosis of small intestine cells. In conclusion, *V. jatamansi* Jones may inhibit RV-induced diarrhea through PI3K/AKT signaling pathway, and could therefore be a potential therapy for diarrhea.

## Introduction

Rotavirus (RV) is the main reason for severe diarrhea among infants and children under 5 years old worldwide [1], especially in developing countries [2]. RV infects enterocytes on the tip of the intestinal villi through a host receptor [3]. Vaccines play anti-RV roles and reduce the incidence of RV infection among children, mitigating the global burden of dehydrating gastroenteritis [4]. However, the introduction of vaccines in developing countries has been limited by economic and environmental conditions [5]. Therefore, the improvement of vaccines and finding new therapies to prevent disease caused by RV are urgently needed.

*Valeriana jatamansi* Jones is a perennial herb mainly found in China and India and can be used as a substitute for European *V. officinalis* [6]). The genus *Valeriana* is well known for its anti-virus and anti-tumor effects [7, 8]. This perennial plant is distributed in Shanxi, Henan, Hunan, Hubei, Sichuan, Guizhou, and Yunnan provinces and the Tibet Autonomous Region, China [9]. The herb is prepared from naturally dried rhizome and root of *V. jatamansi* Jones, which is also widely distributed throughout the temperate Himalayan region [8, 10]. Many phytochemicals from this herb including iridoids, sesquiterpenoids, and essential oils have already been identified. These essential oils are commonly used in India and have both antioxidant and insecticidal activities whereas iridoids and sesquiterpenoids exert moderate neuroprotective effects and inhibitory activity on acetylcholinesterase [12]. However, few studies on *V. jatamansi* Jones have been conducted.

In the present study we investigated the potential role and underlying mechanisms of *V. jatamansi* Jones in RV-induced diarrhea. BALB/c mice and MA104 cells were exposed to RV. CCK-8 and TdT-mediated dUTP nick-end labeling (TUNEL) assay were applied to determine the cytotoxicity and cell apoptosis. Hematoxylin-eosin (HE) staining was performed to determine the effects of *V. jatamansi* Jones on small intestine. *V. jatamansi* Jones could be a potential candidate for diarrhea therapy.

## Materials and Methods

### Cells, Virus and Reagents

MA104 (Rhesus monkey kidney cell line) was purchased from ATCC (USA) and cultured in monolayer cultures using Eagle’s minimal essential medium (Nissui Pharmaceutical, Japan) containing 10% heat-inactivated fetal bovine serum (FBS; Equitech-Bio, USA) and 2 M glutamine in 5% CO_2_ at 37°C. Cells were exposed to RV strain SA11 and harvested after 2 freeze-thaw cycles and stored in aliquots at -80°C. Virus titer was determined by a fluorescent-focus assay with 96-well plates (Corning Incorporated, USA).

*V. jatamansi* was powdered and extracted with 95% ethanol and refluxed. The combined extract was filtered and evaporated (yield 20.7% (w/w)). Albiziae Cortex and Ziziphi Spinosae Semen were powdered, diluted in distilled water, and evaporated with 15.2% (w/w) and 25.8% (w/w) respectively. Junci Medulla was extracted with aqueous ethanol (95%, 1.5 L, v/v) and concentrated (4.5% (w/w)). The extracts were mixed at a ratio of 5:3:5:1 with prepared *V. jatamansi* Jones.

Ribavirin was purchased from Sigma-Aldrich Inc. (USA). Cells were divided into five groups: normal group (without treatment), low group (10 μM *V. jatamansi* Jones), medium group (20 μM *V. jatamansi* Jones), high group (30 μM *V. jatamansi* Jones). The morphological change was observed using a microscope.

### Cell Morphology Study

Cells were seeded into 24-well plates and cultured overnight after RV treatment. Then, cells were washed with PBS, and the morphological changes were observed by light microscopy at at ×100 magnification (Nikon, Japan).

### Cytotoxicity Assay

IC_50_ value was detected with a CCK-8 kit (Dojindo, Japan). Briefly, MA104 cells were plated in 96-well plates at a concentration of 5 × 10^3^ cells/well and then cultured overnight. Next, cells were treated with *V. jatamansi* Jones (0, 1.5, 3, 6, 12, 24, 48 μM) for 48 h. Afterwards, CCK-8 solution was added and incubated for another 2 h. The absorbance values were determined with a microplate reader at the wavelength of 450 nm. The 50% growth inhibition (IC_50_) of *V. jatamansi* Jones was also assessed.

### Quantitative Reverse Transcription (RT)-PCR

Total RNA was extracted from cells using TRIzol method. The extracted RNA was reversely transcribed with a Transcriptor First Strand cDNA Synthesis Kit (Roche Diagnostics GmbH Mannheim, Germany). The PCR was performed using a Real-Time PCR System (Illumina, USA) under the following conditions: 95°C for 10 min, followed by 40 cycles of 95°C for 10 s, 55°C for 30s, and 72°C for 60 s. Results were analyzed with the 2^-ΔΔCt^ method. The mRNA levels were normalized to GAPDH. The primer sequences for Simian RV VP6 [GenBank: L15384.1] were designed as 5’-CTTCTACCAGACGCGGAAAG-3’ (forward) and 5’-ATTCGGCCTGAGAATCACTG-3’ (reverse).

Total RNA was extracted by a single-step isolation procedure using Trizol (Invitrogen, USA). Total RNA were synthesized into cDNA using the Transcriptor First Strand cDNA Synthesis Kit (Roche Diagnostics GmbH). Semi-quantitative RT–PCR was performed to determine expression levels. GAPDH was used as a loading control. All primers were synthesized by SANGON (China). The sequences of the primers used were listed as follows: phosphatidylinositol 3-kinase (PI3K): Forward, 5’-CATCACTTCCTCCT GCTCTAT-3’, Reverse, 5’-CAGTTGTTGGCAATCTTCTTC-3’; protein kinase B (AKT), Forward: 5’-GGACAACCGCCATCCAGACT-3’, Reverse: 5’-GCCAGGGACACCTCCATCTC-3’; GAPDH: Forward, 5’-GGAGCGAGATCCCTCCAAAAT-3’, Reverse, 5’-GGCTGTTGTCATACTTCTCATGG-3’.

### Animals and RV-Induced Diarrhea

Three-day-old suckling specific BALB/c male mice were purchased from the Model Animal Research Center of Nanjing University (China) and housed at 25°C with a 12 h light/dark cycle. The study was approved by the Ethics Committee of Jiangsu Agri-animal Husbandry Vocational College. All the mice were randomly divided into six groups: normal group (Normal, healthy mice orally administered with saline solution), RV infection groups (RV, mice orally administered with RV and without treatment), low-dose group (RV+L, mice orally administered with 10 mg/Kg of *V. jatamansi* Jones) medium-dose group (RV+M, mice orally administered with 20 mg/Kg of *V. jatamansi* Jones), high-dose group (RV+H, mice orally administered with 30 mg/kg of *V. jatamansi* Jones), RV+H+ribavirin group (mice orally administered with 30 mg/Kg of *V. jatamansi* Jones and 60 mg/kg of ribavirin).

*V. jatamansi* Jones or ribavirin administration was performed for 14 days, followed by animal sacrifice, and blood and tissue sample collection.

### Detection of RV in Fecal Specimens

Fecal samples were collected from mice at day post inoculation (DPI1) to DPI7 and homogenized in 0.01 mol/l phosphate-buffered saline (pH 7.2) in a 10% (w/v) solution or suspension and centrifuged at 1200 × g for 20 min. The supernatants were collected and RV shedding was determined through RV antigen detected by PCR according to the manufacturer’s instructions. The test was considered positive if the optical density at 450 nm (OD450) ≥ 0.1.

### Diarrhea Score

The severity of diarrheal illness was assessed by fecal material. Stools were scored from 1 to 4 based on color, texture, and amount. Normal feces were scored 1, exceptionally loose feces scored 2, loose yellow-green feces scored 3, and watery feces scored 4. Stools with a score of ≥ 2 were considered to be diarrhea. The percentage of diarrhea was calculated each day. Mice with no stool were considered as mice with no diarrhea. The diarrhea severity was determined.

### ELISA

The serum levels of interleukin 1β (IL-1β), interferon γ (IFN-γ), IL-6, tumor necrosis factor α (TNF-α), and IL-10, secretory immunoglobulin A (sIgA) were determined with an ELISA kit (China) according to the manufacturer’s instructions. The results were detected with a microplate reader at the wavelength of 450 nm.

### Western Blot

Protein samples of mouse small intestine were extracted by M-PER Mammalian Protein Extraction Reagent (Thermo Scientific, USA) and quantified by Bio-Rad Protein Assay (Bio-Rad, USA) according to the manufacturers’ instructions. The protein samples were separated with 12% sodium dodecyl sulfate polyacrylamide gels and transferred to polyvinylidene fluoride membranes. The membranes were blocked with 5% skimmed milk for 2 h at room temperature and then incubated in rabbit monoclonal primary antibodies for PI3K p85 beta (ab180967, 1: 2,000, Abcam, USA), p-PI3K p85 beta (phospho Y464, ab138364, 1: 1,000, Abcam), AKT (ab179463, 1: 10,000, Abcam), p-AKT (ab38449, 1: 1,000, Abcam) overnight at 4°C. After washing in PBS for 5 times, the blots were incubated in goat anti-rabbit horse-radish peroxidase-conjugated secondary antibody (ab6721, 1: 5,000, Abcam) for 1 h at room temperature. The protein level was determined with an ECL Detection Kit (Thermo Scientific) and analyzed by ImageJ 1.49 software (National Institutes of Health).

### Histologic Examination and Immunohistochemistry

Small intestines were removed and fixed in 4% polyoxymethylene. The slices (5 mm) were secreted and deparaffinized. Then, the slices were stained by hematoxylin and eosin and observed under a light microscope. Crypt depth and villi height were measured with software Image Pro Plus 5.1 (IPP5.1). The villus length was measured from the base to the top of the villi, and the crypt depth was measured between the crypt-villus junction and the base of the crypt.

Tissues were deparaffinized and blocked with 0.3% H_2_O_2_ for elimination of endogenous peroxidase activity. After blocking with goat serum, the slides were incubated with primary antibodies: anti-PI3K p85 beta (ab180967, 1: 2,000, Abcam), anti-p-PI3K p85 beta (phospho Y464, ab138364, 1: 1,000, Abcam), anti-AKT (ab179463, 1: 10,000, Abcam),anti-p-AKT (ab38449, 1: 1,000, Abcam) and then with goat anti-rabbit horse-radish peroxidase-conjugated secondary antibody (ab6721, 1: 5,000, Abcam) at room temperature for 1 h. The image was visualized with a microscope (Nikon).

### TUNEL Assay

Cell apoptosis in the mouse small intestine was detected by TUNEL assay with the In Situ Cell Death Detection Kit (Roche, Switzerland) according to the manufacturer’s instructions. Briefly, the small intestine of each mouse was embedded in paraffin and cut into 5-μm slices. Then, the slices were incubated with Fresh TUNEL mix (50 μl) for 1 h at 37°C and then with converter-POD at 37°C for 30 min. Afterwards, the slices were added with 50 μl of diaminobenzidine (DAB). Then the slices were washed with PBS thrice and then dehydrated, mounted, and captured with an optical microscope (Olympus, Japan). Five fields were randomly chosen for each individual, and apoptotic cells were counted.

### Statistical Analysis

All experiments were performed in triplicate and data were presented as the mean ± deviation standard (SD). Statistical analysis was performed by a one-way ANOVA followed by a Student-Newman-Keul’s test. *p* < 0.05 was considered statistically significant.

## Results

### Effects of RV on Cell Morphology, Cell Proliferation and RV Replication

**Fig. 1:**
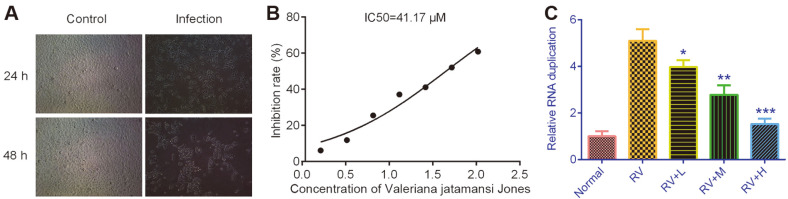
The effects of *Valeriana jatamansi* Jones on cell morphology, RV replication and cell growth. (**A**) MA104 cells were observed under a microscope after RV infection compared with normal cells. (**B**) CCK-8 assay was performed to detect IC_50_ value of *V. jatamansi* Jones on MA104 cells. (**C**) qRT-PCR was performed to evaluate the effects of *V. jatamansi* Jones on RV replication. RV: rotavirus. Normal: healthy mice orally administered with saline solution; RV, mice orally administered with rotavirus and without treatment; RV+L: mice orally administered with rotavirus and 10 mg/kg of *V. jatamansi* Jones, RV+M: mice orally administered with rotavirus and 20 mg/kg of *V. jatamansi* Jones, RV+H, mice orally administered with rotavirus and 30 mg/kg of *V. jatamansi* Jones. *p < 0.05, **p < 0.01 vs. RV group.

As shown in Fig. 1A, the normal cells were distributed evenly and had clear boundaries. After exposure to RV, the cells became swollen and gathered together 24 h later; after 48 h, the cell morphology change was aggravated. Moreover, the IC_50_ value was 41.17 μM for 48 h treatment, indicating that under 41.17 μM, *V. jatamansi* Jones had no significant inhibitory effects on MA104 cell proliferation (Fig. 1B). *V. jatamansi* Jones inhibited RV replication in a dose-dependent manner (Fig. 1C).

### Effects of *V. jatamansi* Jones on PI3K/AKT Signaling Pathway

**Fig. 2:**
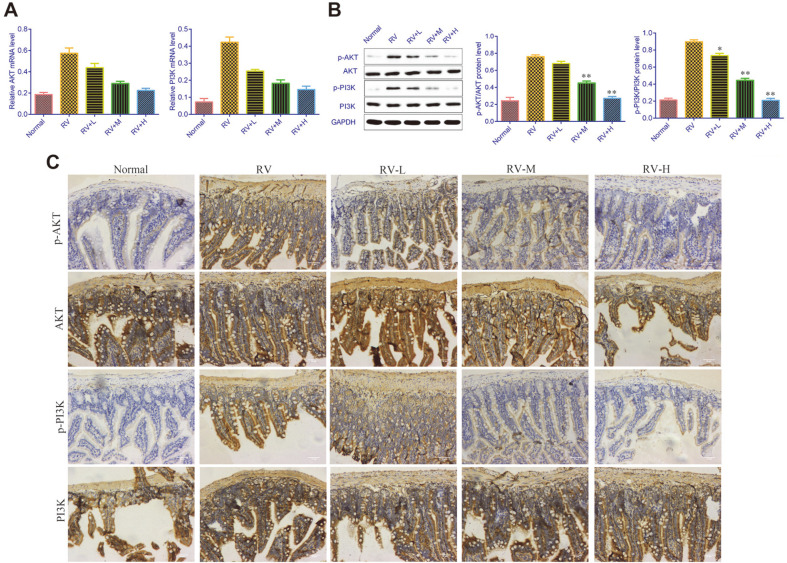
*Valeriana jatamansi* Jones downregulated PI3K/AKT pathways. (**A**) Western blot was performed to detect the mRNA expression in PI3K/AKT signaling pathway. (**B**) Immunohistochemical assay was performed to detect the protein expression in PI3K/AKT signaling pathway. RV: rotavirus; PI3K: phosphatidylinositol-3 kinase; AKT: protein kinase B. Normal: healthy mice orally administered with saline solution; RV, mice orally administered with rotavirus and without treatment; RV+L: mice orally administered with rotavirus and 10 mg/kg of *V. jatamansi* Jones, RV+M: mice orally administered with rotavirus and 20 mg/kg of *V. jatamansi* Jones, RV+H, mice orally administered with RV and 30 mg/kg of *V. jatamansi* Jones. *p < 0.05, **p < 0.01 vs. RV group.

Western blot and immunohistochemical assay were performed to determined the levels of PI3K/AKT pathway. RV treatment upregulated p-PI3K and p-AKT. However, the protein levels of p-PI3K and p-AKT in cells administered with *V. jatamansi* Jones were downregulated in a dose-dependent manner (Fig. 2A). These were paralleled with the results from the immunohistochemical assay (Fig. 2B).

### Effects of *V. jatamansi* Jones on sIgA and Cytokines

**Fig. 3:**
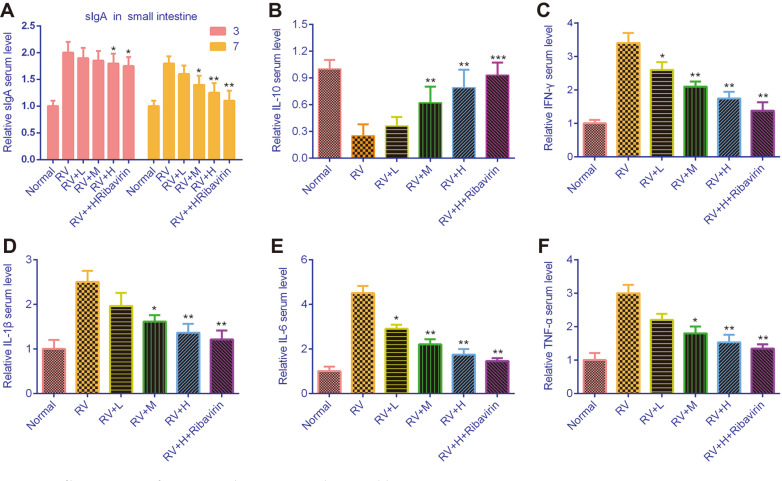
Inflammatory factors and sIgA were detected by ELISA. (**A**) sIgA was detected by ELISA in small intestinal mucous. (**B-F**) The levels of IL-10, IFN-γ, IL-1β, IL-6, and TNF-α in serum were determined by ELISA. RV: rotavirus; sIgA: secretory immunoglobulin A, IL-10: Interleukin 10; IFN-γ: interferon-γ; IL-1β: Interleukin 1β; IL-6: Interleukin 6; TNF-α: Tumor Necrosis Factor Alpha. Normal: healthy mice orally administered with saline solution; RV, mice orally administered with rotavirus and without treatment; RV+L: mice orally administered with rotavirus and 10 mg/kg of *V. jatamansi* Jones, RV+M: mice orally administered with rotavirus and 20 mg/kg of *V. jatamansi* Jones, RV+H, mice orally administered with rotavirus and 30 mg/kg of *V. jatamansi* Jones; RV+H+ribavirin: mice orally administered with rotavirus, 30 mg/kg of *V. jatamansi* Jones and 60 mg/kg of ribavirin. *p < 0.05, **p < 0.01, ***p < 0.001 vs. RV group.

As shown in Fig. 3A, the protein level of sIgA was increased in the diarrhea mice. *V. jatamansi* Jones significantly downregulated sIgA in a dose- and time-dependent manner. Moreover, the expression of pro-inflammatory factors including IL-1β, IFN-γ, IL-6, and TNF-α were significantly increased after RV infection, while anti-inflammatory factor IL-10 was decreased. However, this was reversed by *V. jatamansi* Jones in a dose-dependent manner, which was more potent in high dose of *V. jatamansi* Jones and ribavirin group (Figs. 3B-3F).

### Effects of *V. jatamansi* Jones on RV-Induced Diarrhea Mice Fecal Murine RV Shedding

**Fig. 4:**
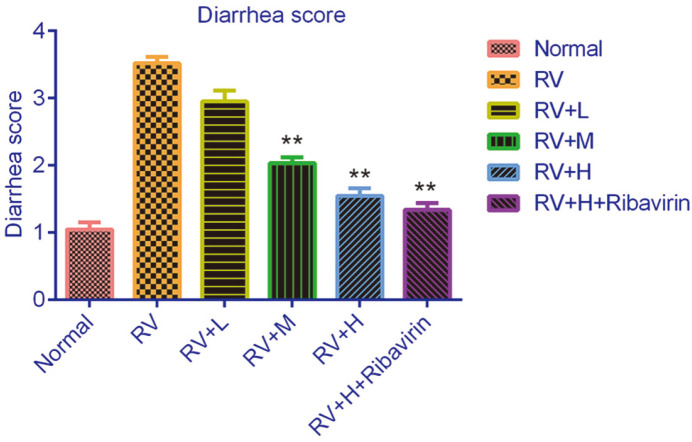
*Valeriana jatamansi* Jones decreased diarrhea scores. To evaluate the therapeutical effects of *V. jatamansi* Jones on RV-induced diarrhea mouse, diarrhea score and RV shedding in stool samples were detected. The diarrhea score for rate-administered different concentrations of *V. jatamansi* Jones and ribavirin. Score of > 2 was considered diarrhea, whereas ≤ 2 was considered normal. RV: rotavirus. Normal: healthy mice orally administered with saline solution; RV, mice orally administered with rotavirus and without treatment; RV+L: mice orally administered with rotavirus and 10 mg/kg of *V. jatamansi* Jones, RV+M: mice orally administered with rotavirus and 20 mg/kg of *V. jatamansi* Jones, RV+H, mice orally administered with rotavirus and 30mg/kg of *V. jatamansi* Jones; RV+H+ribavirin: mice orally administered with rotavirus, 30 mg/kg of *V. jatamansi* Jones and 60 mg/kg of ribavirin. *p < 0.05, **p < 0.01 vs. RV group.

As shown in Fig. 4, normal suckling mice with mock inoculation did not show diarrhea. After exposure to RV, the diarrhea score was significantly increased, suggesting mice with obvious diarrhea. However, the neonatal mice treated with *V. jatamansi* Jones exhibited a decreased diarrhea score in a dose-dependent manner. Moreover, high dose of *V. jatamansi* Jones and ribavirin was more efficacious in decreasing the diarrhea score.

### Effects of *V. jatamansi* Jones on Small Intestine Histopathological Changes and Small Intestine Apoptosis

**Fig. 5:**
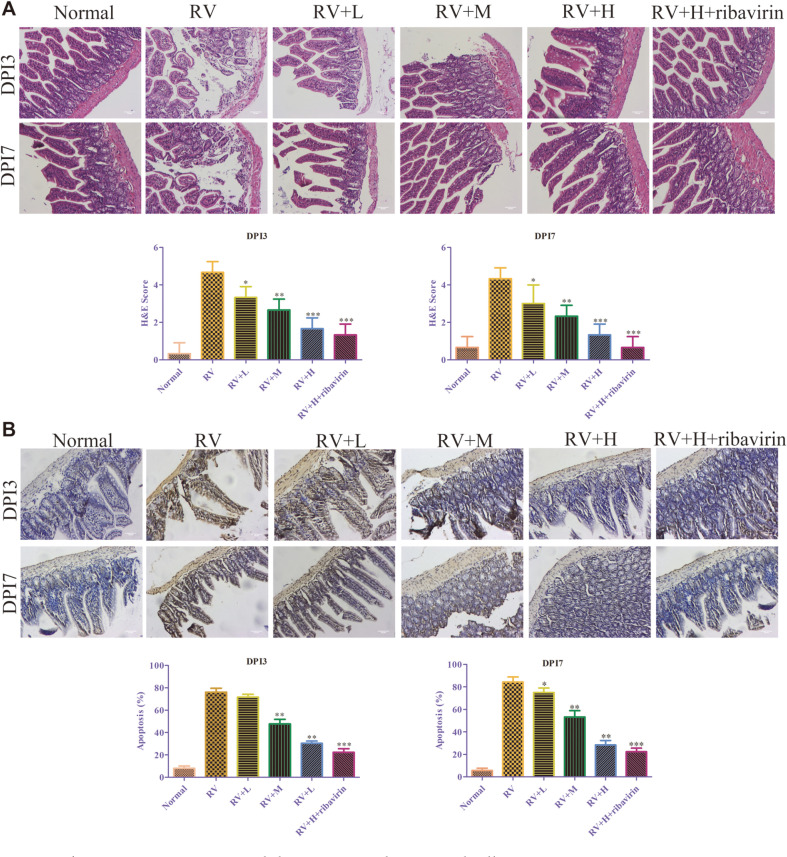
*Valeriana jatamansi* Jones inhibits intestine changes and cell apoptisis. (**A**) Small intestine changes were assessed using HE staining. (**B**) Small intestine apoptosis changes were assessed using TUNEL assay. RV: rotavirus. DPI : day post inoculation. Normal: healthy mice orally administered with saline solution; RV, mice orally administered with rotavirus and without treatment; RV+L: mice orally administered with rotavirus and 10 mg/kg of *V. jatamansi* Jones, RV+M: mice orally administered with rotavirus and 20 mg/kg of *V. jatamansi* Jones, RV+H, mice orally administered with rotavirus and 30 mg/kg of *V. jatamansi* Jones; RV+H+ribavirin: mice orally administered with rotavirus, 30 mg/kg of *V. jatamansi* Jones and 60 mg/kg of ribavirin. *p < 0.05, **p < 0.01, ***p < 0.001 vs. RV group.

HE staining was performed to determine the effects of *V. jatamansi* Jones on small intestine at day 3 and day 7. RV-inoculated mice had significantly swollen villus tips with enterocytes containing large vacuoles, villi atrophy, epithelium defluxion, and crypt hyperplasia. Moreover, virus-inoculated mice showed a thickening of the lamina propria and substantial mononuclear cell infiltration. *V. jatamansi* Jones administration improved the lesion changes and vacuolar degeneration in a dose- and time-dependent manner, which was more efficacious in high dose of *V. jatamansi* Jones and ribavirin group (Fig. 5A).

TUNEL assay was performed to detect the apoptosis of RV-infected small intestine cells. As shown in Fig. 5B, in the RV-induced diarrhea mice, small intestine cell apoptosis was increased compared with the normal mice, which was reversed by *V. jatamansi* Jones. Additionally, high dose of *V. jatamansi* Jones + ribavirin was more efficient in decreasing the apoptosis of small intestine cells induced by RV (Fig. 5B).

## Discussion

Several RV strains such as bovine RV B641 and human Wa RVs have evolved highly sophisticated gene-silencing mechanisms to evade the host-immune response. As the vaccine has not been promoted widely, RV infection often affects healthy infants with high morbidity and mortality, especially in developing countries [13]. MA104 cells are widely used for the growth of animal culture-adapted RV strains [14]. *V. jatamansi* Jones is reported to exert antibacterial activities [15]. However, there is no evidence that *V. jatamansi* Jones inhibits RV infection. In this study, *V. jatamansi* Jones treatment downregulated the expression of RV. Furthermore, the proliferation of MA104 cells infected with RV was suppressed after *V. jatamansi* Jones administration. *V. jatamansi* Jones inactivated PI3K/AKT pathways, and regulated sIgA and cytokines, which was more efficacious in *V. jatamansi* Jones plus ribavirin. These results suggested that *V. jatamansi* Jones may play an anti-RV role in RV-induced diarrhea, which was consistent with a previous study [15]. However, the underlying mechanisms are still unknown.

In previous studies, PI3K/AKT signaling pathways were shown to be intensively involved in the initiation and progression of intestinal diseases [16-18]. Viral infection induces the activation of PI3K/AKT pathways, which, in turn, promotes virus replication [19, 20]. Inactivation of PI3K/AKT pathways inhibits the proliferation of virulent porcine epidemic diarrhea virus and thereby suppresses the proliferation of cells infected with virus [16, 21]. Thus, to block PI3K/AKT pathways may be a promising therapy for virus-infected intestinal diseases. In this study, the expression of p-PI3K/AKT was downregulated by *V. jatamansi* Jones administration. Therefore, *V. jatamansi* Jones may inhibit the proliferation of MA104 cells infected with RV via suppressing RV duplication and inactivating PI3K/AKT pathways. However, the underlying mechanisms are still unclear. RV infection can induce the production of inflammatory cytokines and activation of inflammatory signaling pathways, and promote the development of diarrhea in rats [21, 22]. Importantly, inhibition of PI3K/AKT can reverse the abnormal expression of inflammatory factors and improve the symptoms of diarrhea [23]. Ribavirin plays a crucial role in inactivating PI3K/AKT pathways [24]. Additionally, sIgA is a potential biomarker for porcine epidemic diarrhea virus (PEDV) [25]. Therefore, inactivating PI3K/AKT pathways to regulate sIgA and the cytokines (such as IFN-γ, IL-1β, TNF-α, IL-6, and IL-10), which participate in inflammation response, may be a therapeutic target for RV-induced diarrhea. In this study, *V. jatamansi* Jones reversed the increase of sIgA induced by RV in a dose- and time-dependent manner, which was more potent after combined treatment of *V. jatamansi* Jones (30 mg/kg) and ribavirin. Additionally, *V. jatamansi* Jones plus ribavirin was more potent in regulating cytokines involved in inflammation response. These results suggested blocking PI3K/AKT signaling pathways with *V. jatamansi* Jones may be an efficient therapy for RV-induced diarrhea.

Moreover, this study also evaluated the effect of *V. jatamansi* Jones on RV in vivo. Diarrhea score results suggested *V. jatamansi* Jones and ribavirin improved diarrhea symptoms in RV-induced mice.

Collectively, *V. jatamansi* Jones played an anti-virus and protective role in RV-induced diarrhea through inactivating PI3K/AKT pathways. The combination of *V. jatamansi* Jones and ribavirin was more potent in inhibiting RV-induced diarrhea. *V. jatamansi* Jones could be used as a new drug and serve as a novel strategy for RV-induced diarrhea.
