# Corrigendum to: *Valeriana jatamansi* Jones Inhibits Rotavirus-Induced Diarrhea via Phosphatidylinositol 3-Kinase/Protein Kinase B Signaling Pathway

**DOI:** 10.4014/jmb.2024.3402.484

**Published:** 2024-02-28

**Authors:** Bin Zhang, Yan Wang, Chunmao Jiang, Caihong Wu, Guangfu Guo, Xiaolan Chen, Shulei Qiu

**Affiliations:** 1 Jiangsu Agri-Animal Husbandry Vocational College, Taizhou, Jiangsu 225300, P.R. China; 2 Food, Animal and Plant Inspection and Quarantine Technical Center of Shanghai Customs, Shanghai 210000, P.R. China

In the article titled “*Valeriana jatamansi* Jones Inhibits Rotavirus-Induced Diarrhea via Phosphatidylinositol 3-Kinase/Protein Kinase B Signaling Pathway”, the authors noticed that there were several picture misuses in this paper. Misused pictures are listed below: picture p-AKT/Normal, AKT/Normal, AKT/RV, AKT/RV-L, AKT/RV-M, AKT/RV-H, PI3K/RV-L and PI3K/RV-M in Fig. 2C immunohistochemical figures; picture DPI3/RV and DPI7/RV in Fig. 5A HE staining figures; picture DPI7/Normal and DPI7/RV+H+ribavirin in Fig. 5B TUNEL staining figures. The main reason for the pictures to be misused was that the sequence number of the notes in the pictures was disordered when the test company submitted the results. To ensure the reliability of the results, we retested the samples. The updated Fig. 2C, Fig. 5A and Fig. 5B are as follows:

In addition, we found two other problems. The first problem is that the second paragraph of the result is not comprehensive. Another problem is the incomplete illustration of Figure 2. The result description and legend of Figure 2 are updated as follows:

**Effects of *V. jatamansi* Jones on PI3K/AKT Signaling Pathway**

RT-PCR, western blot and immunohistochemical assay were performed to determined PI3K/AKT pathway. RV treatment upregulated p-PI3K and p-AKT. However, mRNA levesl and the protein levels of p-PI3K and p-AKT in cells administered with *V. jatamansi* Jones were downregulated in a dose-dependent manner (Fig. 2A and Fig. 2B). These were paralleled with the results from the immunohistochemical assay (Fig. 2C).

**Fig. 2. *Valeriana jatamansi* Jones downregulated PI3K/AKT pathways.**

(A) RT-PCR was performed to detect the mRNA expression in PI3K/AKT signaling pathway. (B) Western blot was performed to detect the protein expression in PI3K/AKT signaling pathway. (C) Immunohistochemical assay was performed to detect the protein expression in PI3K/AKT signaling pathway. RV: rotavirus; PI3K: phosphatidylinositol-3 kinase; AKT: protein kinase B. Normal: healthy mice orally administered with saline solution; RV, mice orally administered with rotavirus and without treatment; RV+L: mice orally administered with rotavirus and 10 mg/kg of *V. jatamansi* Jones, RV+M: mice orally administered with rotavirus and 20 mg/kg of *V. jatamansi* Jones, RV+H, mice orally administered with RV and 30 mg/kg of *V. jatamansi* Jones. *p<0.05, **p<0.01 vs. RV group.

## Figures and Tables

**Fig. 2C:**
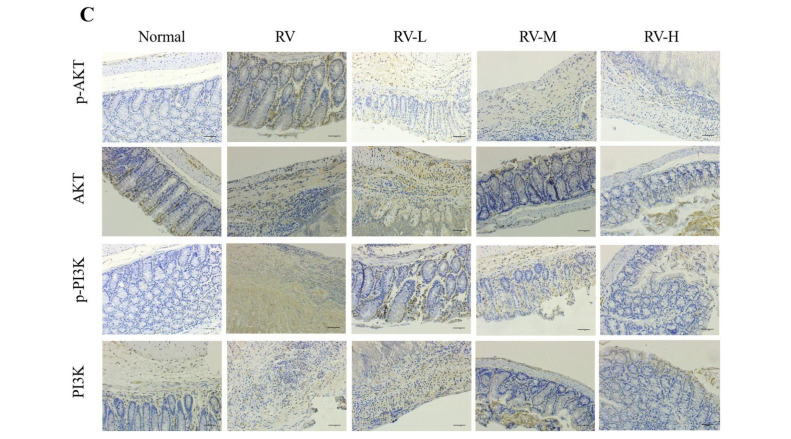
Immunohistochemical assay was performed to detect the protein expression in PI3K/AKT signaling pathway.

**Fig. 5A:**
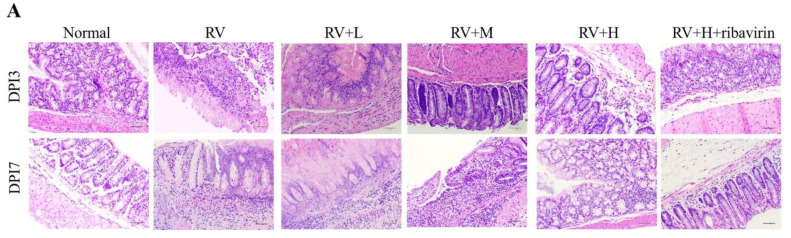
Small intestine changes were assessed using HE staining.

**Fig. 5B:**
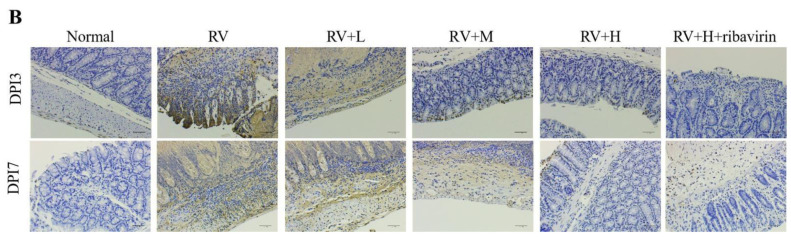
Small intestine apoptosis changes were assessed using TUNEL assay.

